# Postoperative endophthalmitis treatment with antibiotics associated or not with pars plana vitrectomy: a randomized clinical trial

**DOI:** 10.1186/s40942-025-00640-1

**Published:** 2025-02-18

**Authors:** Vinicius Campos Bergamo, Luis Filipe Nakayama, Nilva Simeren Bueno de Moraes, Ivan Maynart Tavares, Mauro Silveira De Queiroz Campos, Ana Luisa Hofling-Lima, Maurício Maia

**Affiliations:** 1 Retina Division, Department of Ophthalmology, Vitreoretinal Surgery, Escola Paulista de Medicina, Universidade Federal de São Paulo, 806, Botucatu Street, São Paulo, 04026-062 Brazil; 2 Glaucoma Division, Department of Ophthalmology, Escola Paulista de Medicina, Universidade Federal de São Paulo, São Paulo, Brazil; 3 Cornea and External Diseases Division, Department of Ophthalmology, Escola Paulista de Medicina, Universidade Federal de São Paulo, São Paulo, Brazil; 4 Laboratory of Ocular Microbiology Division, Department of Ophthalmology, Escola Paulista de Medicina, Universidade Federal de São Paulo, São Paulo, Brazil

**Keywords:** Pars plana vitrectomy, Endophthalmitis, Intravitreal antibiotic injection, Endophthalmitis vitrectomy study

## Abstract

**Background:**

Postoperative endophthalmitis (PSE) is a severe ocular complication that can lead to irreversible vision loss or even globe atrophy. The Endophthalmitis Vitrectomy Study (EVS) historically guided PSE management but is increasingly questioned due to advances in pars plana vitrectomy (PPV) techniques and its narrow focus on cataract surgery. This study aimed to compare PPV followed by intravitreal antibiotic injection at the end of surgery (PPV + IVAIES) with intravitreal antibiotic injection alone (IVAI) in managing PSE.

**Methods:**

This randomized clinical trial included 35 pseudophakic patients with PSE following cataract extraction, anti-vascular endothelial growth factor (anti-VEGF) injections, or glaucoma surgeries. Participants were randomized to receive either PPV + IVAIES (n = 12) or IVAI (n = 23). Best-corrected visual acuity (BCVA) was assessed at baseline and days 7, 30, 60, and 90 post-intervention. Clinical worsening, defined as lack of improvement or progression of symptoms within 48–72 h, guided retreatment protocols. Group A (PPV + IVAIES) received repeat IVAI if required, while Group B (IVAI) underwent delayed PPV with repeat IVAI. Statistical significance was assessed using repeated measures ANOVA and logistic regression.

**Results:**

Both groups showed significant BCVA improvement (p < 0.001). PPV + IVAIES resulted in faster recovery, with superior BCVA at day 7 (p = 0.019) and day 30 (p = 0.041). Retreatment was required in 39.1% of the IVAI group but not in the PPV + IVAIES group (p = 0.015). Subgroup analysis indicated a trend toward better early outcomes with early PPV (p = 0.029).

**Conclusions:**

Early PPV + IVAIES provides faster visual recovery and reduces retreatment rates compared to IVAI alone. Multicenter studies are warranted to confirm these findings and refine clinical guidelines.

*Trial registration* ClinicalTrials.gov identifier: NCT04192994.

## Background

Postoperative endophthalmitis (PSE) is a severe condition that can lead not only to irreversible decrease of vision but also to global atrophy and death and as such requires prompt treatment [1]. The pivotal Endophthalmitis Vitrectomy Study (EVS) provided clinical criteria that have guided the management of this condition for decades [2]. This very important study demonstrated that pars plana vitrectomy (PPV) was beneficial for patients with a best-corrected visual acuity (BCVA) of light perception at the initial presentation and that treatment with intravitreal antibiotics alone (IVAI) should be more effective for patients with better levels of initial BCVA.

However, considering that the EVS was conducted several decades ago, before the advancements in current PPV techniques, including 23-gauge (G) and 25-G instrumentation, 20,000 cuts/minute vitreous cutters, improved and safer surgical tools, and wide-angle viewing systems, the EVS recommendations recently have been questioned increasingly [3–7].

The EVS focused exclusively on cataract surgery, and due to the lack of large clinical trials, its findings have been broadly applied to guide the management of other types of postoperative endophthalmitis, including intraocular infection after intravitreal injections, which recently has become increasingly common [8, 9]. However, emerging evidence supports the value of early PPV in managing cases of PSE [10, 11].

While the EVS has significantly influenced the management of endophthalmitis for decades, emerging evidence has demonstrated the benefits of early PPV in these cases. Additionally, a key limitation of the EVS is its narrow focus on post-cataract surgery endophthalmitis, thereby excluding other etiologies of the condition [2–11]. In light of these controversies, the current randomized clinical trial aims to provide further evidence on the role of early PPV to manage PSE compared to IVAI.

## Methods

This randomized controlled trial was conducted at the Vitreoretinal Unit of the Ophthalmology Department at the Escola Paulista de Medicina, Universidade Federal de São Paulo, Brazil. The study spanned from January 2019 to December 2022 and is registered on ClinicalTrials.gov under the identifier NCT04192994.

This study was conducted as a prospective randomized clinical trial (RCT), and informed consent was obtained from all participants. The trial was approved by the institutional ethics committee (approval number 209/2019) and adhered to the principles of the Declaration of Helsinki.

### Study participants

Patients aged 18 years or older who presented with clinical signs of acute postoperative endophthalmitis (PSE) within 6 weeks of cataract extraction, intravitreal anti-vascular endothelial growth factor (VEGF) injections, PPV, and glaucoma surgery were included in the study. Eligibility was determined based on the clinical history, including symptoms such as ocular hyperemia, pain, and inflammation in the anterior chamber and vitreous. The exclusion criteria included a BCVA of no light perception (LP) at the initial presentation, previous treatment for PSE, endogenous or traumatic causes of endophthalmitis, endophthalmitis secondary to infectious keratitis, or the presence of corneal infiltration or melting at the time of presentation and vitrectomized eyes.

The patients underwent four follow-up visits in addition to the initial evaluation, with comprehensive ophthalmologic examinations and visual acuity (VA) assessments conducted at each visit. These assessments were performed on day 0 (baseline) and subsequently on days 7, 30, 60, and 90 post-intervention. BCVA was assessed using standardized ETDRS charts at 4 m.

The BCVA was converted to the logarithm of the minimum angle of resolution (logMAR). For patients qualitatively reported to have counting fingers, hand motions, light perception (LP), or no LP vision, logMAR values of 1.80, 2.30, 2.80, and 3.00 were assigned, respectively.

A total of 51 patients were screened; 16 were excluded (11 declined participation, 5 did not meet inclusion criteria), resulting in 35 participants for analysis. Due to the pandemic, some patient follow-up was compromised, with missed visits or infection by SARS-CoV-2, leading to their exclusion from the final analysis.

The triggering factors of PSE were categorized into three groups: post-intravitreal anti-VEGF injections, post-cataract extraction, and other causes, including bleb-related and vitrectomy surgeries.

Bevacizumab (Avastin, Genentech, South San Francisco, CA, USA) was the anti-VEGF agent of primary choice, with vials prepared using the pooling method.

The chosen randomization method was simple 1:1 randomization.

### Surgical procedures

For the first group of patients who underwent PPV and IVAI at the end of surgery (group A, PPV + IVAIES), PPV was performed using 23-G instruments. Vitreous samples were collected using a 23-gauge vitrectomy probe, with 0.5–1.0 mL samples sent for microbiological analysis.

A conservative approach was adopted, with attempts to detach the posterior hyaloid only when feasible, and careful shaving of the vitreous base was performed. Vitreous samples were collected at the beginning of the surgery using a vitrectomy probe before irrigation. Sclerotomies were closed with 7–0 Vicryl sutures. At the end of the surgery, intravitreal injections of 0.05 mL vancomycin 1.0 mg and 0.05 mL ceftazidime 2.25 mg were administered. The vitreous substitute in all surgeries was Balanced Salt Solution (BSS). During follow-up, patients received topical moxifloxacin 0.3% for 7 days, and prednisolone acetate 1% gradually tapered. No patients experienced any complications, and no oral antibiotics were prescribed.

For the second group (group B, IVAI), treatment consisted of an IVAI alone. The tap-and-inject technique was performed in the operating room using a blepharostat, following international protocol standards [12]. The procedure included the administration of 5% povidone-iodine drops, collection of vitreous samples (0.1 mL were collected using a 25-gauge needle and syringe) and culturing of samples for bacteria and/or fungus, and intravitreal injection of 0.05 mL of vancomycin 1 mg and 0.05 mL of ceftazidime 2.25 mg. No oral or intravitreal corticosteroids were administered.

The decision to retreat was made 48 to 72 h after the primary intervention. Eyes with clinical worsening from baseline were advised to undergo retreatment, following the following options. Patients in group A underwent isolated IVAI (since PPV had been previously performed); and those in group B were advised to undergo PPV along with repeat IVAI.

### Outcome measures

The primary endpoints were the final BCVA on day 90 and the mean change in the BCVA at each time interval across the groups. Secondary endpoints included the reintervention rate between the two groups.

To assess the impact of delayed PPV on outcomes, a subgroup analysis was performed to compare the outcomes of eyes that underwent immediate PPV (group A) with those that underwent delayed PPV (group B with reintervention).

### Statistical analysis

Analyses were conducted using SPSS software version 20.0 (IBM, SPSS Inc, Chicago, IL, USA) and STATA version 17 (StataCorp LLC, College Station, TX, USA). Categorical variables were summarized with absolute and relative frequencies, while numerical variables were described using the mean, quartiles, minimum, maximum, and standard deviation. To assess associations among categorical variables, we used the chi-square test or Fisher's exact test, and identified local differences using standardized adjusted residuals, with values above 1.96 indicating significant associations.

Comparisons between two groups were made using the Student's t-test, and for more than two groups, analysis of variance (ANOVA) was applied. Both tests assume normal data distribution; when this assumption was violated, the Mann–Whitney test was used as a non-parametric alternative.

The correlations between numerical variables were evaluated using Pearson's correlation, or Spearman's correlation if the data were non-normally distributed.

The effect of interventions on VA over time was assessed using repeated measures ANOVA, with data normality checked via the Kolmogorov–Smirnov test. Due to sample size constraints, exact logistic regression was applied to evaluate the impact of interventions and demographic or clinical characteristics on VA outcomes.

P < 0.05 was considered significant throughout the analyses.

## Results

Of the 35 participants, 42.9% were men, and 57.1% were women. The mean age was 61.9 ± 12.3 years (range 18–84). Diabetes prevalence was 28.6%, with no cases of HIV or immunosuppressive drug use.

**Fig. 1:**
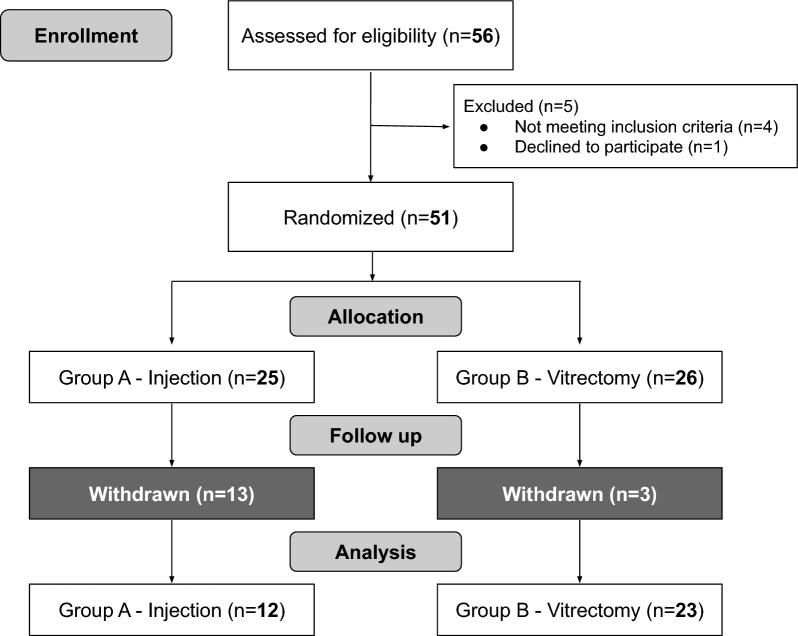
Flowchart of the randomized clinical trial evaluating the management of postoperative endophthalmitis (PSE). A total of 56 patients were assessed for eligibility, with 5 excluded for not meeting inclusion criteria or declining participation. Fifty-one patients were randomized into two groups: Group A (Intravitreal Antibiotic Injection—IVAI, n = 25) and Group B (Pars Plana Vitrectomy with Intravitreal Antibiotic Injection at the End of Surgery—PPV + IVAIES, n = 26). Following withdrawals during follow-up, the final analysis included 12 patients in Group A and 23 in Group B

The baseline characteristics in Tables 1 and 2 summarize the bacterial resistance profiles of the positive cultures samples. Figure 1 shows the diagram for participant enrollment (Fig. 1).

**Table 1:** Patients Characteristics by Intervention

	Intervention		Total	p
	IVAI (N = 23, 65.7%)	PPV (N = 12; 34.3%)		
Sex, n(%)				0.537^a^
Female	14/23 (60.9)	6/12 (50.0)	20/35 (57.1)	
Male	9/23 (39.1)	6/12 (50.0)	15/35 (42.9)	
Age (years)				0.089^c^
Media ± SD	59.3 ± 13.5	66.8 ± 8.1	61.9 ± 12.3	
Median (IIQ)	60.0 (54.0–68.0)	67.0 (59.3–70.8)	63.0 (58.0–68.0)	
Eye, n(%)				0.713^a^
Right	13/23 (56.5)	6/12 (50.0)	19/35 (54.3)	
Left	10/23 (43.5)	6/12 (50.0)	16/35 (45.7)	
Endophthalmitis cause, n(%)				0.871^b^
ANTI-VEGF INJECTION	8/23 (34.8)	5/12 (41.7)	13/35 (37.1)	
CATARACT EXTRACTION	13/23 (56.5)	6/12 (50.0)	19/35 (54.3)	
OTHER	2/23 (8.7)	1/12 (8.3)	3/35 (8.6)	
Time before intervention				0.342^c^
Media ± SD	3.8 ± 2.4	3.1 ± 1.7	3.6 ± 2.2	
Median (IIQ)	3.0 (2.0–5.0)	2.0 (2.0—4.5)	3.0 (2.0—5.0)	
Culture positivity, n(%)				0.076^a^
No	13/23 (56.5)	3/12 (25.0)	16/35 (45.7)	
Yes	10/23 (43.5)	9/12 (75.0)	19/35 (54.3)	
Bacteria^1^, n(%)				0.315^b^
*Klebsiella aerogenes*	1/10 (10.0)	0/9 (0.0)	1/19 (5.3)	
*Staphylococcus aureus*	2/10 (20.0)	1/9 (11.1)	3/19 (15.8)	
*Staphylococcus coagulase negativa*	3/10 (30.0)	1/9 (11.1)	4/19 (21.1)	
*Staphylococcus epidermidis*	2/10 (20.0)	6/9 (66.7)	8/19 (42.1)	
*Staphylococcus haemolyticus*	1/10 (10.0)	0/9 (0.0)	1/19 (5.3)	
*Streptococcus alfa-hemol oralis do gr viridans*	0/10 (0.0)	1/9 (11.1)	1/19 (5.3)	
*Streptococcus alfa-hemol pneumoniae*	1/10 (10.0)	0/9 (0.0)	1/19 (5.3)	
Retreat, n(%)				**0.015**^**b**^
No	14/23 (60.9)	12/12 (100.0)	26/35 (74.3)	
Yes	9/23 (39.1)	0/12 (0.0)	9/35 (25.7)	

^1^Only for positive cases

p—descriptive level of Chi-square test(^a^), Fisher's Exact test(^b^), Student's t-test(^c^)

SD: Standard Deviation

**Table 2:** Bacterial resistance profile by patient intervention

Patient	Endophthalmitis causes	Bacteria	Retreat	Oxa	Vanco	Cipro	Moxi	Genta	Tobra	Amica	Azitro	Eritro	Cefox	Ceftaz	Ceftr	Line	Peni
1	AVASTIN	Streptococcus alfa-hemol pneumoniae	NO	N	S	N	N	N	N	N		N	N	S	N		N
2	AVASTIN	*Klebsiella* *aerogenes*	NO	N	N	S	S	S	S	S		N	N	N	S		N
3	AVASTIN	*Staphylococcus* *epidermidis*	NO	S	N	I	S	S	S	S		S	N	N	N		N
4	AVASTIN	*Staphylococcus* *epidermidis*	NO	S	S	S	S	S	S	S	S		S	N	N	S	N
5	AVASTIN	*Staphylococcus* coagulase negativa	NO	**R**	S	**R**	**R**	S	S	S	S		**R**	N	N	N	**R**
6	AVASTIN	*Staphylococcus* *aureus*	NO	S	S	S	S	S	S	S	**R**		S	N	N	N	**R**
7	AVASTIN	*Staphylococcus* *epidermidis*	NO	S	N	I	S	S	S	S		R	N	N	N		N
8	AVASTIN	*Staphylococcus* *epidermidis*	**YES**	**R**	S	**R**	**R**	S	S	N	N	N	**R**	N	N	N	**R**
9	AVASTIN	*Staphylococcus* *epidermidis*	NO	S	N	**R**	**R**	**S**	**R**	S		R	N	N	N		N
10	AVASTIN	*Staphylococcus* *epidermidis*	NO	S	N	**R**	**R**	**S**	**R**	S		S	N	N	N		N
11	CATARACT	*Staphylococcus* coagulase negativa	NO	S	S	I	S	S	S	S		R	N	N	N		N
12	CATARACT	*Staphylococcus* coagulase negativa	NO	S	S	S	S	S	S	S	S		S	N	N	N	**R**
13	CATARACT	*Staphylococcus* *haemolyticus*	**YES**	**R**	S	**R**	**R**	S	S	S	**R**		**R**	N	N	N	**R**
14	CATARACT	*Staphylococcus* *aureus*	NO	S	N	**R**	S	S	S	S		R	N	N	N		**R**
15	CATARACT	*Staphylococcus* *coagulase* *negativa*	NO	S	N	I	N	S	S	S		S	N	N	N		N
16	CATARACT	*Streptococcus* *alfa*-*hemol* oralis do gr viridans	NO	N	S	N	N	N	N	N	**R**		N	N	S	N	S
17	CATARACT	*Staphylococcus* *epidermidis*	NO	**R**	S	**R**	**R**	**R**	**R**	S		N	N	N	N		N
18	OTHER	*Staphylococcus* *aureus*	NO	S	N	I	S	S	**R**	S		S	N	N	N		**R**
19	OTHER	*Staphylococcus* *epidermidis*	NO	**R**	S	**R**	**R**	S	I	S	**R**		**R**	N	N	N	N

Twelve eyes were assigned to the PPV + IVAIES group (12/35, 34.3%) and 23 eyes to the IVAI group (23/35, 65.7%). Among the patients, 13 developed PSE as a result of intravitreal bevacizumab injections (13/35, 37.1%), 19 had a cataract extraction (19/35, 54.3%), and three had other causes (3/35, 8.6%). No patients experienced any complication, and no oral antibiotics were prescribed.

The initial baseline BCVA was 2.18 ± 0.67 in the IVAI group and 2.00 ± 0.77 in the PPV group, a difference that did not reach significance (p = 0.636).

**Fig. 2:**
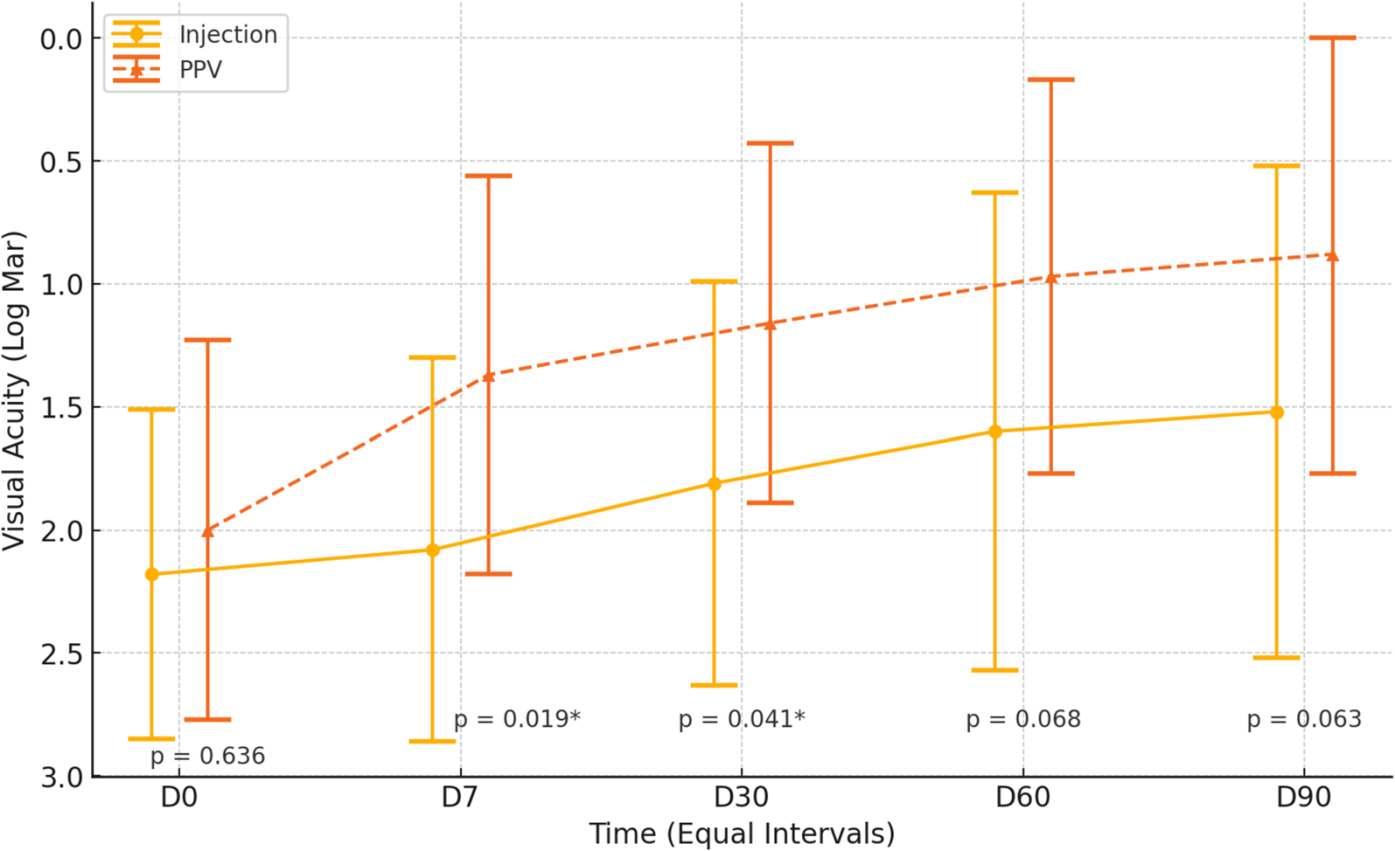
Changes in visual acuity over time in patients treated with intravitreal antibiotic injection (IVAI) versus pars plana vitrectomy with intravitreal antibiotic injection at the end of surgery (PPV + IVAIES). The y-axis represents best-corrected visual acuity (BCVA) in logMAR, where lower values indicate better vision. The x-axis indicates follow-up intervals (baseline—D0, and days 7, 30, 60, and 90 post-intervention). The dashed orange line represents the PPV + IVAIES group, while the solid yellow line represents the IVAI group. Statistically significant differences in BCVA between groups were observed at days 7 (p = 0.019) and 30 (p = 0.041), but not at later intervals. Error bars represent standard deviations

During the follow-up period, the VAs in both groups improved significantly compared to the initial VA (Fig. 2).

Figure 2 and Table 3 show the VAs over time between the IVAI group and the PPV + IVAIES group.

**Table 3:** Summary measures of visual acuity by intervention and evaluation time—overall

	Visual acuity (logMAR ± SD)					ANOVA		
	D0	D7	D30	D60	D90	Intervention	Time	Interaction Intervention × Time
Intervention						0.039	< 0.001	0.161
IVAI	2.18 ± 0.67	2.08 ± 0.78	1.81 ± 0.82	1.60 ± 0.97	1.52 ± 1.00			
PPV	2.00 ± 0.77	1.37 ± 0.81	1.16 ± 0.73	0.97 ± 0.80	0.88 ± 0.89			
*p-value*	0.636	0.019*	0.041*	0.068	0.063			

logMAR: logarithm of the minimum angle of resolution

*statistically significant are intentionally highlighted

SD: standard deviation; IVAI: Intravitreal antibiotic injection; PPV: Pars plana vitrectomy

Table 3 shows the mean BCVAs by group and evaluation time, along with the descriptive levels of the repeated-measures ANOVA, which assesses the effect of time, group, and the interaction between group and timelines. The presence of an interaction indicated that the means of the groups evolved differently over time. However, no interaction effect between time and group was observed, indicating that the mean variations were not distinct between the groups (p = 0.161). Thus, a time effect was observed in both intervention groups (p < 0.001); post-hoc tests under the estimated model indicated that, on average, the preoperative BCVA was higher (worse BCVA) than the BCVA at days 30, 60, and 90 postoperatively, which were similar to each other. In addition, the mean BCVA at day 7 postoperatively was worse than at days 60 and 90 postoperatively, which were also similar to each other. Moreover, on average, the BCVA of the group that underwent IVAI (group B) was worse than that of the PPV group (group A) at all evaluation times (p = 0.039).

**Fig. 3:**
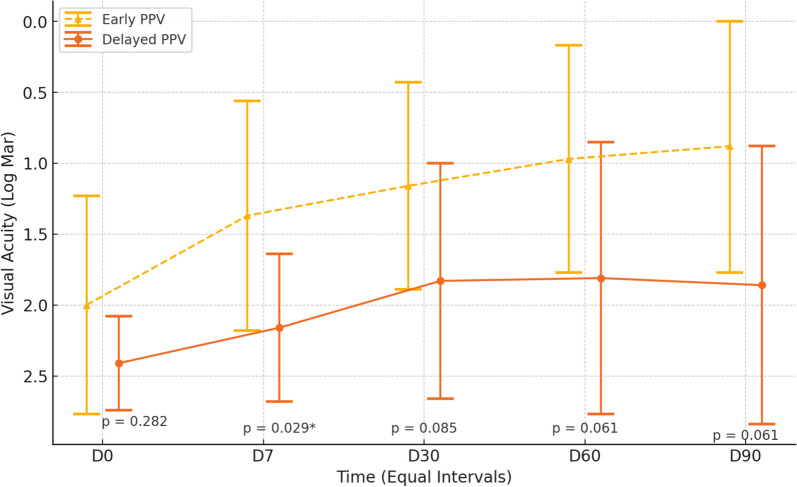
Comparison of visual acuity improvement over time between early pars plana vitrectomy (PPV) and delayed PPV in the management of postoperative endophthalmitis (PSE). The y-axis shows best-corrected visual acuity (BCVA) in logMAR, where lower values indicate better vision. The x-axis represents follow-up intervals (baseline—D0, and days 7, 30, 60, and 90 post-intervention). The dashed yellow line represents early PPV, while the solid orange line represents delayed PPV. A statistically significant difference in BCVA was observed at day 7 (p = 0.029), favoring early PPV. Error bars indicate standard deviations

After the first intervention, a reintervention was required within the first 48 h in nine patients, all of whom were in the IVAI group (9/23; 39.1%). Figure 3 and Table 4 show the BCVA over time between the eyes that underwent PPV as the primary management or retreatment. Positive cultures were not obtained from any eyes that underwent a reintervention.

**Table 4:** Summary measures of VA by intervention and evaluation time—patients who underwent vitrectomy

	Visual Acuity (logMAR ± SD)					ANOVA		
	Initial	D7	D30	D60	D90	Intervention	Time	Interaction Intervention × Time
Intervention						0.019	< 0.001	0.429
Early PPV	2.00 ± 0.77	1.37 ± 0.81	1.16 ± 0.73	0.97 ± 0.80	0.88 ± 0.89			
Delayed PPV	2.41 ± 0.33	2.16 ± 0.52	1.83 ± 0.83	1.81 ± 0.96	1.86 ± 0.98			
p-value	0.282	0.029*	0.085	0.061	0.061			

logMAR: logarithm of the minimum angle of resolution; *statistically significant are intentionally highlighted; SD: standard deviation; PPV: Pars plana vitrectomy

No interaction effects between time and group were observed (Fig. 3, Table 4), indicating that the mean variations were not distinct between the groups (p = 0.429). However, a time effect was observed in both intervention groups (p < 0.001); post-hoc tests under the estimated model indicated that, on average, the preoperative BCVA was higher (worse BCVA) than at all subsequent time points, which were similar to each other. Furthermore, at all evaluation times, the mean BCVA of the group that underwent a retreatment (late vitrectomy) was higher (worse) than that of the patients who underwent the initial vitrectomy (p = 0.019).

No associations were found among the clinical characteristics considered and retreatment (Table 5).

**Table 5:** Distribution of demographic and clinical characteristics of patients by retreatment

	Retreat		Total	p
	No (N = 14, 60.9%)	Yes (N = 9, 39.1%)		
Sex, n(%)				1.000^a^
Female	9/14 (64.3)	5/9 (55.6)	14/23 (60.9)	
Male	5/14 (35.7)	4/9 (44.4)	9/23 (39.1)	
Age (years)				0.342^b^
Media ± SD	61.5 ± 15.7	55.9 ± 8.9	59.3 ± 13.5	
Median (IIQ)	66.0 (56.3 to 70.0)	60.0 (47.0–62.5)	60.0 (54.0 to 68.0)	
VA pre—logMar				0.195^b^
Media ± SD	2.0 ± 0.8	2.4 ± 0.3	2.2 ± 0.7	
Median (IIQ)	2.3 (1.7–2.8)	2.3 (2.3–2.8)	2.3 (2.0–2.8)	
Previous surgery, n(%)				1.000^a^
Anti-VEGF injection	5/14 (35.7)	3/9 (33.3)	8/23 (34.8)	
Cataract extraction	8/14 (57.1)	5/9 (55.6)	13/23 (56.5)	
Other	1/14 (7.1)	1/9 (11.1)	2/23 (8.7)	
Time before intervention				0.802^c^
Media ± SD	3.9 ± 2.5	3.7 ± 2.3	3.8 ± 2.4	
Median (IIQ)	3.0 (2.0–5.3)	2.0 (2.0–6.0)	3.0 (2.0–5.0)	
Culture positivity, n(%)				0.197^a^
No	6/14 (42.9)	7/9 (77.8)	13/23 (56.5)	
Yes	8/14 (57.1)	2/9 (22.2)	10/23 (43.5)	

^a^p—descriptive level of Chi-Square test (^a^), Fisher's Exact test (^b^) and Student's t-test (^c^); SD: standard deviation; logMAR: logarithm of the minimum angle of resolution

**Fig. 4:**
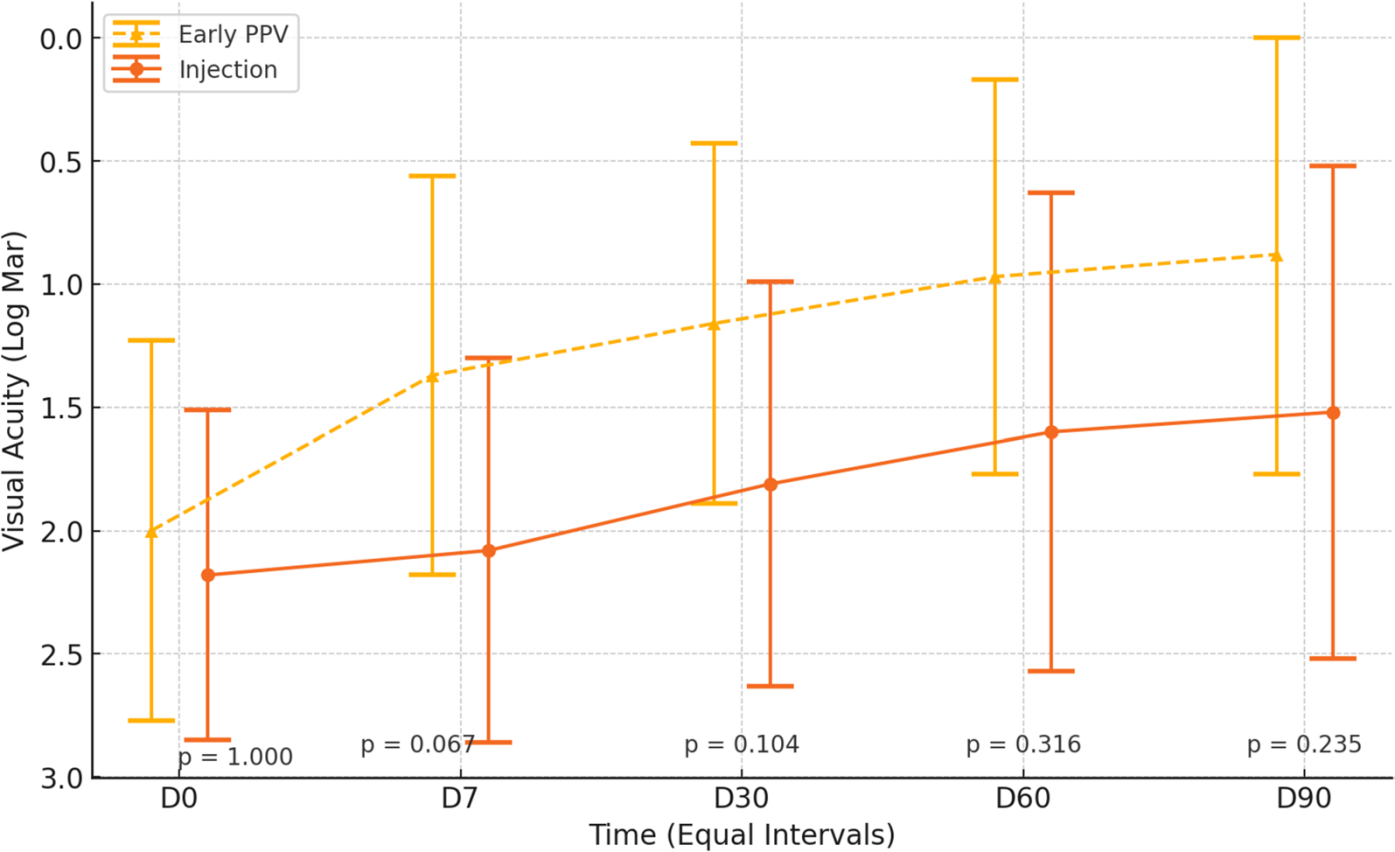
Visual acuity improvement over time in patients treated with early pars plana vitrectomy (PPV) compared to intravitreal antibiotic injection (IVAI) alone. The y-axis represents best-corrected visual acuity (BCVA) in logMAR, where lower values indicate better vision. The x-axis represents follow-up intervals (baseline—D0, and days 7, 30, 60, and 90 post-intervention). The dashed yellow line corresponds to the early PPV group, while the solid orange line represents the IVAI group. No statistically significant differences in BCVA were observed at any time point (p > 0.05). Error bars indicate standard deviations

Figure 4 and Table 6 illustrate the VAs over time for eyes that underwent only one treatment procedure.

**Table 6:** Summary measures of VA by intervention and evaluation time—PPV and IVAI without retreatment

	Visual acuity (logMAR ± SD)					ANOVA		
	Initial	D7	D30	D60	D90	Intervention	Time	Interaction Intervention x Time
Intervention						0.166	< 0.001	0.115
IVAI	2.04 ± 0.79	2.03 ± 0.92	1.78 ± 0.86	1.42 ± 0.99	1.24 ± 0.96			
Early PPV	2.00 ± 0.77	1.37 ± 0.81	1.16 ± 0.73	0.97 ± 0.80	0.88 ± 0.89			
p-value	1.000	0.067	0.104	0.316	0.235			

logMAR: logarithm of the minimum angle of resolution; SD: standard deviation; IVAI: Intravitreal antibiotic injection; PPV: Pars plana vitrectomy

No interaction effect between time and group was observed, indicating that the mean variations were not distinct between the groups (p = 0.115) (Table 6). However, a time effect was observed in both intervention groups (p < 0.001); post-hoc tests under the estimated model indicated that, on average, the preoperative BCVA was higher (worse BCVA) than on days 30, 60, and 90 postoperatively, which were similar to each other. In addition, the mean BCVA on day 7 postoperatively was higher (worse) than on days 60 and 90 postoperatively, which were also similar to each other. Moreover, no differences in the mean BCVA were observed between the groups at all evaluation times (p = 0.166).

An association was observed only between the type of intervention and retreatment (p = 0.015) (Table 7); the IVAI group had a higher percentage of retreatments compared to the PPV group (39.1% versus 0.0%).

**Table 7:** Retreatment rates by intervention group (IVAI vs. PPV + IVAIES)

Retreat n (%)	Group IVAI	Group PPV + IVAIES	*p* = *0.015*
No	14/23 (60.9)	12/12 (100.0)	26/35 (74.3)
Yes	9/23 (36.1)	0/12 (0.0)	9/35 (25.7)

PPV: Pars Plana Vitrectomy; IVAI: Intravitreal Antibiotic Injection; IVAIES: Intravitreal Antibiotic Injection at End of Surgery

## Discussion

Before the publication of the EVS in 1995, the number of studies on endophthalmitis was limited, with no clinical trials available and a lack of evidence on how to effectively manage the condition [13]. Over time, especially after the pandemic, the number of publications on endophthalmitis has increased significantly, with the infection emerging as a growing trend in recent years [14]. The findings and implications of the EVS have remained a focal point of discussion, continuing to be a hot topic in the field [3, 4, 15, 16].

Our findings suggested that while both treatment modalities resulted in significant BCVA improvement over time, PPV + IVAIES provided superior outcomes regarding the BCVA improvement and lower retreatment rates compared to IVAI alone.

The results from the current study agreed with previous reports in the literature that emphasize the importance of early vitrectomy in cases of PSE [17, 18]. The EVS [2], which historically guided treatment decisions, primarily focused on cataract surgery cases and included no cases related to intravitreal injections, which recently have become more prevalent. As a result, the EVS recommendations may not fully apply to contemporary scenarios in which PPV techniques have advanced significantly.

Recently, Sen et al. [5] reported the role of PPV versus IVAI in eyes after cataract extraction with a presenting BCVA better than hand motions in cases of endophthalmitis. The authors concluded that PPV resulted in earlier recovery, although the final VAs were similar between the groups.

The current randomized clinical trial aimed to compare the outcomes of PPV followed by intravitreal antibiotic injection at the end of surgery versus intravitreal antibiotic injection alone to manage acute PSE, including eyes that had undergone cataract extraction, anti-VEGF injections, bleb-related surgeries, and PPV.

Although the two final groups evaluated in this study did not have the same number of eyes, the baseline characteristics (Table 1) showed no significant differences, which strengthens and adds relevance to the study.

Table 1 showed a vitreous culture positivity rate of 54.3%, which is lower than the EVS [2], but consistent with more recent publications [19, 20]. Among the positive cultures, coagulase-negative *Staphylococcus* was present in more than 60%, with *S. epidermidis* accounting for 42.1%. *Streptococcus spp*. were present in 10% of the sample. Cioana et al.[21] described an evolutionary difference between *Staphylococci* and *Streptococci* agents, with the former showing better BCVA outcomes than the latter; however, these differences were not observed in the present study. When comparing the positivity rates of vitreous samples collected using different techniques, the PPV group showed a higher positivity rate than the IVAI group (75% vs. 43.5%), with a marginally significant difference likely attributed to the small sample size of the study (p = 0.076). These results agree with previous studies that reported a higher positivity rate when biopsy is performed during vitrectomy [22].

Table 2 provides an overview of the bacterial susceptibility profiles of culture-positive samples. In all cases of cataract surgery, patients received intracameral moxifloxacin at the conclusion of the procedure. Despite this prophylaxis, five patients developed endophthalmitis. Among these, five isolates were resistant to both oxacillin and moxifloxacin, with two cases necessitating a secondary vitrectomy for treatment. Endophthalmitis also was observed in cases with oxacillin-susceptible and moxifloxacin-susceptible isolates, suggesting that intracameral prophylaxis alone may have been insufficient to prevent intraocular infection. The vancomycin susceptibility analysis indicated its potential effectiveness as monotherapy for controlling gram-positive infections.

One patient who developed endophthalmitis after a bevacizumab injection did not receive intraocular prophylaxis beyond the standard intravitreal injection protocol.

Two endophthalmitis cases were attributed to *Streptococcus spp*., one after a bevacizumab injection and the other associated with cataract surgery.

When analyzing the baseline VAs in the IVAI and PPV + IVAIES groups, we observed that no clinical parameters justified a difference in the initial vision. However, by day 7, a significant difference emerged in favor of the eyes that underwent PPV + IVAIES (p = 0.019). This difference persisted through day 30 (p = 0.041), but by days 60 and 90 (p = 0.068 and p = 0.063, respectively), the VA curves between the groups converged, and the statistical difference disappeared (Fig. 2, Table 3). This can be explained by the ability of PPV to remove the cloudy vitreous, promoting greater optical clarity, which allows for faster visual recovery. This suggests that the removal of infected vitreous material and the early intervention with PPV can significantly reduce the burden of infection and inflammatory mediators, leading to better visual recovery. Thus, as described by Sen et al. [5], we see that the initial visual gain is much faster with PPV + IVAIES.

During the follow-up, retreatment was required in nine patients in the IVAI group and no patients in the PPV + IVAIES group (9/23, 39.1%; 0/12, 0.0%) (p = 0.015) (Table 7). This finding may suggest that early intervention with PPV + IVAIES may prevent the need for retreatment due to therapeutic failure, as reported by Zhao et al.[22] These findings highlight a potential limitation of the tap-and-inject approach, especially in cases in which the infection might be more widespread or when the initial intervention is insufficient to control the infection. In addition, the lower retreatment rate in the PPV group further underscores the efficacy of this surgical approach in managing PSE.

Furthermore, for comparison purposes, all eyes that underwent PPV + IVAIES were divided into two groups (early PPV and delayed PPV), and their BCVAs after treatment were compared; the BCVA on day 7 was significantly better in the early PPV group (p = 0.029) (Fig. 3, Table 4), supporting previous findings that PPV promotes faster visual improvement. This difference was not observed on days 30, 60, or 90. These subgroup analyses highlighted that early intervention is crucial for optimal outcomes. Eyes that underwent delayed PPV showed poorer visual outcomes, suggesting that the timing of surgical intervention plays a critical role in the prognosis of PSE.

Table 5 shows the characteristics of the patients who underwent retreatment. No differences were seen between the patients with therapeutic failure and those who underwent only one surgical procedure. Angelia et al.[23] recently published a systematic review and meta-analysis, which cited poor prognostic factors in cases of endophthalmitis, including low initial BCVA, presence of a positive culture, and age over 85 years. None of these characteristics were present in the retreatment group.

Thus, we speculated that the cause of retreatment may be based on the decision to administer an antibiotic injection rather than performing a PPV.

In a further comparison, we isolated the retreatment variables and compared only the groups that had one intervention (PPV + IVAIES vs. IVAI without retreatment). The BCVAs on days 0, 7, 30, 60, and 90 did not differ significantly, but the PPV group exhibited a slight tendency toward better vision (Fig. 4, Table 6).

The results of this study demonstrated that both treatment groups (PPV + IVAIES and IVAI) showed significant improvement in BCVA over time. However, the early differences observed between the groups, favoring PPV + IVAIES within the first 30 days, converged by days 60 and 90, resulting in similar visual outcomes at the end of follow-up.

This convergence in BCVA outcomes suggests that, while PPV + IVAIES facilitates faster visual recovery, the long-term benefits may be comparable to IVAI, provided that clinical worsening is promptly managed with appropriate interventions. These findings highlight the importance of considering the timeframe of visual recovery as a key factor in treatment decisions, particularly for patients requiring rapid visual rehabilitation to resume daily activities.

The observed convergence could be attributed to the gradual resolution of infection and inflammation in both groups, regardless of the initial treatment modality. Although, the significantly higher retreatment rate in the IVAI group (39.1% vs. 0%) underscores a potential limitation of this approach, suggesting that PPV + IVAIES may provide superior control of infection and inflammatory burden, thereby reducing the risk of therapeutic failure.

Despite the clear advantages of PPV shown in this study, it is important to acknowledge certain limitations. The sample size was relatively small, which may limit the generalizability of our findings. In addition, the study was conducted at one center, which could introduce bias related to specific clinical practices or patient demographics. Future multicenter studies with larger sample sizes should validate these findings and provide more definitive guidance on the management of PSE.

In conclusion, performing early PPVs was associated with significantly better and faster BCVA recovery and lower retreatment rates compared to IVAI. Although a trend toward improved long-term BCVA was observed in the PPV group, this difference did not reach significance. These findings suggest that early surgical intervention with PPV provides substantial benefits in the management of PSE, particularly in reducing the risk of therapeutic failure. Further multicenter studies are required to validate these results.

## Data Availability

No datasets were generated or analysed during the current study.

## Abbreviations

PSE: Postoperative endophthalmitis
EVS: Endophthalmitis vitrectomy study
PPV: Pars Plana vitrectomy
IVAIES: Intravitreal antibiotic injection at the end of surgery
IVAI: Intravitreal antibiotic injection
BCVA: Best-corrected visual acuity
logMAR: Logarithm of the minimum angle of resolution
VEGF: Vascular endothelial growth factor
G: Gauge
VA: Visual acuity
BSS: Balanced salt solution
