# Correction: Postoperative endophthalmitis treatment with antibiotics associated or not with pars plana vitrectomy: a randomized clinical trial

**DOI:** 10.1186/s40942-025-00647-8

**Published:** 2025-02-27

**Authors:** Vinicius Campos Bergamo, Luis Filipe Nakayama, Nilva Simeren Bueno de Moraes, Ivan Maynart Tavares, Mauro Silveira De Queiroz Campos, Ana Luisa Hofling‑Lima, Maurício Maia

**Affiliations:** 1 Retina Division, Department of Ophthalmology, Vitreoretinal Surgery, Escola Paulista de Medicina, Universidade Federal de São Paulo, 806, Botucatu Street, São Paulo, 04026-062 Brazil; 2 Glaucoma Division, Department of Ophthalmology, Escola Paulista de Medicina, Universidade Federal de São Paulo, São Paulo, Brazil; 3 Cornea and External Diseases Division, Department of Ophthalmology, Escola Paulista de Medicina, Universidade Federal de São Paulo, São Paulo, Brazil; 4 Laboratory of Ocular Microbiology Division, Department of Ophthalmology, Escola Paulista de Medicina, Universidade Federal de São Paulo, São Paulo, Brazil

**Correction: Int J Retin Vitr 11**,** 18 (2025)**

10.1186/s40942-025-00640-1

Following the publication of the original article [1], the authors identified an error in the **Methods** section. The original text incorrectly stated that informed consent was waived due to the retrospective nature of the study. However, this study was conducted as a **prospective randomized clinical trial (RCT)**, and **informed consent was obtained from all participants** before enrollment. The corrected sentence should read as follows:

**Original text (incorrect)**:

*“Although this was an RCT*,* informed consent was waived due to its retrospective nature*,* as approved by the institutional ethics committee (approval number 209/2019).”*

**Corrected text**:

*“This study was conducted as a prospective randomized clinical trial (RCT)*,* and informed consent was obtained from all participants. The trial was approved by the institutional ethics committee (approval number 209/2019) and adhered to the principles of the Declaration of Helsinki.”*

The original article [1] has been updated.
